# Effect of physical exercise in bariatric surgery patients: protocol of a randomized controlled clinical trial

**DOI:** 10.1186/s13063-021-05056-4

**Published:** 2021-02-01

**Authors:** Andrea Herrera-Santelices, Andrea Tabach-Apraiz, Karen Andaur-Cáceres, Antonio Roberto Zamunér

**Affiliations:** 1 Department of Physical Medicine and Rehabilitation, San Juan de Dios Hospital, Curicó, Chile; 2 Laboratory of Clinical Research in Kinesiology, Department of Kinesiology, Universidad Católica del Maule, Talca, Chile; 3 Department of Nutrition and Dietetics, San Juan de Dios Hospital, Curicó, Chile

**Keywords:** Physical exercise, Bariatric surgery, Morbid obesity

## Abstract

**Background:**

Bariatric surgery is an effective approach to weight loss and long-term comorbidity resolution. Although recommended in several guidelines, supervised exercise has not been systematically prescribed after bariatric surgery. The aim of this study is to determine the effects of two types of exercise, moderate-intensity continuous training (MICT) and high-intensity interval training (HIIT), on body composition, cardiopulmonary function, and perceived quality of life in bariatric surgery patients.

**Methods:**

This randomized controlled exploratory pilot trial will include 75 adults of both sexes scheduled for bariatric surgery. They will be randomly assigned to one of three groups: (1) MICT, (2) HIIT, or (3) a control group. The intervention will occur 2 days a week for 4 months. Outcomes will be assessed at four points: (1) 1 week before surgery, (2) 21 days after surgery (baseline before the exercise program), (3) 8 weeks after beginning the exercise program, and (4) 1 week after the end of intervention. Primary outcomes will include body composition, heart rate variability, and 6-min walk test and quality of life scores. Secondary outcomes will be maximal respiratory pressure, flowmeter, hand dynamometry, and 30-s sit-to-stand test results.

**Discussion:**

Both exercise protocols in this study were developed according to evidence-based practice. It is expected that, after 16 weeks of intervention, body composition (measured by electrical bioimpedance), cardiopulmonary function (measured by heart rate variability, maximal inspiratory pressure, maximal expiratory pressure, peak expiratory flow, handgrip strength, and the 6-min walk test), and perceived quality of life (measured by the Moorehead-Ardelt quality of life questionnaire II and bariatric analysis and reporting outcome system scores) will improve, especially in the HIIT group.

**Trial registration:**

ClinicalTrials.gov NCT04235842. Registered on 22 January 2020.

## Administrative information

**Table:** 

Title {1}	Effect of physical exercise in bariatric surgery patients: protocol of a randomized controlled clinical trial.
Trial registration {2a and 2b}.	ClinicalTrials.gov, NCT04235842, registered 22 January 2020.
Protocol version {3}	January 21, 2020. Version 2.
Funding {4}	No Funding
Author details {5a}	**Andrea Herrera-Santelices (AHS):** Universidad Católica del Maule, Talca, Chile. Servicio de Medicina Física y Rehabilitación, Hospital San Juan de Dios, Curicó, Chile. **Andrea Tabach-Apraiz (ATA):** Servicio de Medicina Física y Rehabilitación, Hospital San Juan de Dios, Curicó, Chile. **Karen Andaur-Cáceres (KAC):** Servicio de Nutrición, Hospital San Juan de Dios, Curicó, Chile. **Antonio Roberto Zamunér (ARZ):** Departamento de Kinesiología, Universidad Católica del Maule, Talca, Chile.
Name and contact information for the trial sponsor {5b}	Not applicable, this trial does not have sponsor.
Role of sponsor {5c}	Not applicable, this trial does not have sponsor.

## Introduction

### Background and rationale {6a}

Obesity is an abnormal state of health characterized by excess body fat due mainly to caloric intake and expenditure imbalance [1]. The daily energy consumption of this population is estimated at approximately 3000 Kcal/day, far above the requirements of sedentary individuals [2].

In its latest National Health Survey (2017), Chile was found to have a high risk of non-communicable diseases and a few protective factors. A total of 10,301,529 Chileans (58.6% of the population) has some degree of excess weight, of whom 39.8% are overweight, 31.2% are obese, and 3.2% are morbidly obese.

Studies have shown [3–5] that obesity leads to a state of chronic inflammation and oxidative stress, which is related to numerous chronic diseases such as cardiovascular and respiratory diseases [6, 7]. Obese people also suffer from social pressure, leading to impaired social interaction, body image rejection and low self-esteem, which compromises their psychological health, well-being and, thus, negatively impacts their quality of life [8, 9].

When morbidly obese patients do not respond to the usual obesity treatments, such as exercise, dieting, and pharmacological treatment, they become candidates for restrictive and/or malabsorptive bariatric surgery [10, 11]. Currently, four procedure types exist: adjustable gastric band, Roux-en-Y gastric bypass, vertical sleeve gastrectomy, and bilio-pancreatic bypass [12, 13].

Patients usually experience significant weight loss in the first postoperative year (56 to 85% of their excess weight), of which about 70% is fat mass and 30% lean mass [14]. Some studies have shown that bariatric surgery results not only in weight loss and comorbidity changes, but also in complications such as severe anemia, osteopenia, dehydration, constipation, intestinal obstruction, vitamin and mineral deficiency, fatigue, and posture changes [15, 16]. Preventing such complications is crucial for bariatric surgery patients, especially through non-pharmacological means.

Thus, exercise could be a relevant solution since it maintains muscle mass, generates tension and load on the bones, activates the metabolism, red blood cell production and intestinal peristalsis, improves cardiopulmonary function and general functionality, contributes to bone mineralization, and helps maintain independence [17]. Indeed, several studies have shown the benefits of regular exercise before and after bariatric surgery [18–20].

Among bariatric surgery patients, moderate-intensity continuous training (MICT) effectively decreases postoperative lean mass loss and increases fat mass loss, controls glucose homeostasis, and improves cardiovascular capacity [20–22]. High-intensity interval training (HIIT) has recently been proposed as an alternative to MICT and is well tolerated by overweight and insulin-resistant individuals [23, 24]. The HIIT method has proven effective in improving cardiorespiratory fitness, maximal oxygen uptake (VO_2_max), and insulin sensitivity in healthy subjects [25–27]. In addition, it has been described as more motivating than MICT due to a greater feeling of fatigue and its shorter sessions [28]. However, to our knowledge, no studies have compared the effectiveness of MICT and HIIT in postoperative bariatric surgery patients.

Although participation in regular physical activity programs is a standard recommendation after bariatric surgery in Chile, supervised exercise is not currently recommended or prescribed. Therefore, clarifying the effects of MICT and HIIT on body composition, cardiopulmonary function, and quality of life after bariatric surgery is relevant, especially in our local context.

### Objectives {7}

This study will aim to determine the effect of MICT and HIIT programs on body composition, cardiopulmonary function, and perceived quality of life in bariatric surgery patients. We will also compare the effects of supervised exercise programs with those of standard follow-up. We hypothesized that both exercise programs would lead to greater improvement in the assessed outcomes than standard recommendations. Moreover, we expect the HIIT protocol to result in greater gains than MICT.

### Trial design {8}

This protocol is a randomized pilot exploratory study, single-blind, three-arm, parallel-group study. Participants will be randomly assigned to one of the three groups: (1) a control group (CG), (2) a moderate-intensity continuous exercise training group (MICT-G), or (3) a high-intensity interval exercise training group (HIIT-G).

## Methods: participants, interventions, and outcomes

### Study setting {9}

The study will be carried out at the Department of Physical Medicine and Rehabilitation Service in the San Juan de Dios Hospital, a public hospital in the city of Curicó, located at Chacabuco # 121 street, in the Maule region, Chile. All procedures will be conducted by at least two experienced physiotherapists and the department is strategically located to ensure that any medical emergencies can be promptly cared for. All procedures will be performed in a room with controlled temperature (22–24 °C) and air humidity (40–60%).

### Eligibility criteria {10}

#### Inclusion criteria

Participants of either sex will be considered eligible if they are between 18 and 65 years old, have undergone bariatric surgery at the above-mentioned hospital, have been cleared for exercise, are in the final phase of operative wound healing, have been administered antithrombotics after surgery, and have no plans to change their residence in the year after surgery.

#### Exclusion criteria

Participants with immediate postoperative complications (anastomosis or wound dehiscence) or decompensated comorbidities, who are on dialysis, or who suffer from a neuromotor disease will be excluded.

### Who will take informed consent? {26a}

After receiving a list of patients scheduled for bariatric surgery from a staff member of Bariatric Surgery Service of the hospital, AHS will telephone each patient, invite them to participate, and make an individual appointment. In this, the objective of the study, inclusion and exclusion criteria, participation, risks, benefits, and ethical implications will be explained. In the case that the potential participant wants to participate in the research, the informed consent will be signed in 3 copies: one for the participants, one for the researcher, and one for de clinical record from the hospital.

### Additional consent provisions for collection and use of participant data and biological specimens {26b}

Not applicable, this trial does not have biological specimens.

## Interventions

### Explanation for the choice of comparators {6b}

Participants will be randomly assigned to one of the three groups: (1) a control group (CG) that will receive standardized recommendations, (2) a moderate-intensity continuous exercise training group (MICT-G), or (3) a high-intensity interval exercise training group (HIIT-G).

### Intervention description {11a}

Participants allocated to the MICT and HIIT groups will perform two weekly sessions of supervised exercise for 16 weeks. Sessions will be divided into a 3-week adaptation period and 13 weeks of formal training. All sessions will consist of:

- Joint mobility (3 min): head and neck, shoulder, elbow, wrist, hip, knee, ankle, and trunk.
- Warm-up (7 min): treadmill walking at 1–2 km/h.
- Aerobic component:
  MICT group: 15 min on a cycle ergometer at moderate intensity (40–60% of heart rate reserve) or at a perceived exertion rating of 5–6 out of 10.
  HIIT group: 8 min on a cycle ergometer, including a 60-s sprint at 90% maximal heart rate followed by a 60-s rest until four sets are completed.
- Strength training: 15 min of exercise in the large muscle groups with weight machines or dumbbells, including 1–2 sets of 8–10 repetitions at 50–60% of one-repetition maximum strength (1RM).
- Cool-down (5 min): proprioceptive neuromuscular facilitation exercises for abdominal muscles and the pelvic floor will be performed on a mat. Stretching exercises for arm, leg, and trunk muscles will be performed in three 30-s sets. Hemodynamic variables will be checked to ensure that blood pressure and heart rate values return to baseline.

#### Exercise progression

Beginning with the fourth week, the aerobic load in the MICT group will be adjusted to 60% of the heart rate reserve and the duration will be adjusted to 30 min. In the HIIT group, the number of sets will be increased to 1 by week until 10 sprints. Strengthening exercises will be performed in 3 sets of 10–12 repetitions, with intensities between 60 and 70% 1RM. The 1RM value will be assessed at baseline and week 8.

During both training programs, heart rate, blood pressure, oxygen saturation, and perceived exertion will be evaluated with the Borg CR-10 scale.

Participants allocated to the CG will be following the indications of regular physical activity practice according to WHO (at least 150 min per week of moderate physical activity or at least 75 min of intense physical activity), which will be explained by ATA. The protocol followed by all patients operated of bariatric surgery at the San Juan de Dios hospital is to receive these indications.

### Criteria for discontinuing or modifying allocated interventions {11b}

Will be criteria for discontinuing: participant request, severe alteration of hemodynamic parameters during the training, participants who attend less than 85% of the training sessions, participants who were absent on evaluation days, and participants from the training group who engage other regular physical exercise.

### Strategies to improve adherence to interventions {11c}

Not applicable, this trial does not have strategies to improve adherence.

### Relevant concomitant care permitted or prohibited during the trial {11d}

Not applicable, this trial does not have concomitant care permitted or prohibited.

### Provisions for post-trial care {30}

Once the study is completed, the CG and group with less effect of the study will be invited to carry out the training that has obtained the best effect.

### Outcomes {12}

Outcomes will be assessed at four time points: (1) 1 week before surgery, (2) 21 days after surgery (baseline before starting the exercise program), (3) 8 weeks after the beginning of the exercise program, and (4) 1 week after the end of intervention.

Primary outcomes will be (1) body composition, (2) heart rate variability, (3) 6-min walk test results, and (4) perceived quality of life. Secondary outcomes include the results for (1) maximal respiratory pressure, (2) flowmeter, (3) hand dynamometry, (4) the 30-s sit-to-stand test, and (5) the basal metabolic rate.

Data collection will include sex, age, place of residence, marital status, education level, and physical activity level according to the International Physical Activity Questionnaire [29].

#### Body composition

Body composition will be measured with tetrapolar bioelectrical impedance, (INBODY 270, Inbody Co. Ltd., Korea). The body fat percentage, muscle and bone mass, and basal metabolic rate will be evaluated.

#### Heart rate variability

R-R intervals will be recorded with a Polar V800 heart rate monitor (Polar, Oi, Finland); a sensor will be placed on the chest at the fifth intercostal space. The participant will then rest in the supine position for 10 min to stabilize heart rate and blood pressure. R-R intervals will be recorded under the following conditions: (1) after resting in the supine position for 10 min and (2) after resting in the orthostatic position for 10 min (active standing). The participants’ respiratory rate will be recorded throughout the test.

Heart rate variability will be analyzed through spectral analysis in an autoregressive model. The spectral components will be obtained at low frequency (LF, 0.04–0.15 Hz) and high frequency (HF, 0.15–0.4 Hz) bands in absolute units (ms^2^). Standardized units are calculated as the ratio between LF or HF (absolute units) and the power spectral density, minus the very low frequency component (VLF, 0.003–0.04 Hz) and multiplied by 100. The LF band is modulated by the sympathetic and parasympathetic autonomic nervous system (with sympathetic predominance), the HF band is associated with cardiac vagal control, and the LF/HF ratio is calculated to assess sympathovagal balance [30].

#### Maximal respiratory pressures

Maximal inspiratory (MIP) and expiratory (MEP) pressure will be assessed with a respiratory pressure meter (MicroRPM, MicroMedical Ltd., Kent, UK). All measurements will be performed with the participant seated, using a 2-mm aperture mouthpiece and a nose clip to prevent air leakage. MIP will be measured from residual volume, whereas MEP will be measured from total lung capacity. Clear instructions about performing the test will be provided [31].

#### Flowmeter

While the patient is seated, a mini-Wright flowmeter (Clement Clarke, Mason, OH, USA), nosepiece, and disposable nozzle will be used to measure peak expiratory flow. Peak expiratory flow measurement will be based on maximum inspiration. After being instructed the use of the flowmeter, the patients will be asked to blow as hard and as long as possible [32].

#### Hand dynamometry

To evaluate handgrip strength, a digital dynamometer will be used (CAMRY EH101, Guangdong, China). After being instructed about the test and use of the dynamometer, the participants will perform a maximal isometric contraction for 5 s with each hand while standing [33].

#### Six-minute walk test

The 6-min walk test will be used to evaluate functional capacity. It will take place in a flat corridor with a 15-m track. A cone will be placed at each meter to determine the beginning and ending distance. Participants will be instructed to walk back and forth as quickly as possible for 6 min, and the total distance covered during will be recorded. Before and after the test, blood pressure, heart rate, and oxygen saturation values will be measured. Verbal encouragement will be given every minute, according to Mexican National Institute of Respiratory Disease guidelines [34].

#### Perceived quality of life

The Moorehead-Ardelt quality of life questionnaire II will be applied at each evaluation, and the Bariatric Analysis and Reporting Outcomes System (BAROS), which has been validated for bariatric surgery patients, will be applied at the final evaluation [35, 36].

#### Moorehead-Ardelt quality of life questionnaire II

This test measures quality of life in 6 dimensions: self-esteem, physical activity, social activity, work activity, and sexual activity. Each dimension has 10 response options that are accompanied by images for clarification. Each answer is scored through a visual scale ranging from − 0.5 (most unfavorable situation) to + 0.5 (most favorable situation). The sum of all dimensions produces a global score: very good (2.1 to 3), good (1.1 to 2), fair (− 1 to 1), poor (− 2 to − 1.1), and very poor (− 3 to − 2.1).

#### Bariatric analysis and reporting outcomes system

This test measures quality of life and the benefits of bariatric surgery by incorporating the weight loss or gain percentage after surgery into the Moorehead-Ardelt questionnaire, including comorbidity resolution, reoperation, and complications. While the Moorehead-Ardelt scoring is identical to that described above, the other items are scored according to dimension. Scores for the weight loss dimension (as percent of excess weight lost) were as follows: − 1 (0–24%), 0 (25–49%), 1 (50–74%), and 2 (75–100%). Scores for the comorbidities dimension were as follows: − 1 (aggravated), 0 (no changes), 1 (some improvement), 2 (one major comorbidity resolved and improvement in others), and 3 (all major comorbidities resolved and all others improved). Scores for the complications dimension were as follows: deduct 0.2 points for a minor complication, deduct 1 point for a major complication, and deduct 1 point for reoperation. Global scores for the instrument are the sum of all dimensions, categorized as failure (≤ 1), fair (> 1 to 3), good (> 3 to 5), very good (> 5 to 7), and excellent (> 7 to 9).

#### Thirty-second sit-to-stand test

Participants will be instructed to cross their arms over their chest and stand in front of a reinforced 44-cm-high chair that is positioned against a wall. They will then sit and stand as many times as possible in 30 s, and the number of repetitions will be recorded.

### Participant timeline {13}

**Fig. 1:**
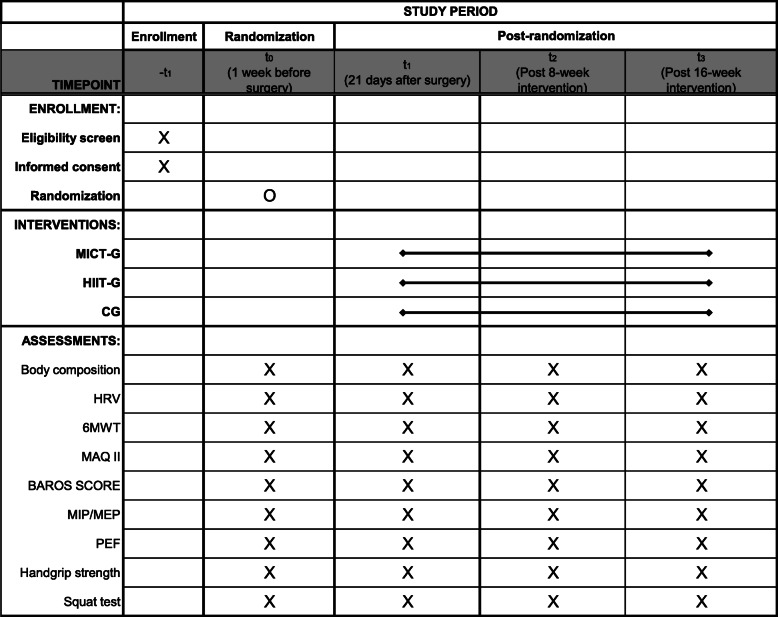
Recommended SPIRIT figure with the participant timeline

Figure 1 show the recommended SPIRIT figure with the participant timeline.

### Sample size {14}

GPower 3.1 was used to determine the sample size, assuming a power of 80% and a significance level of 5%. Calculations were based on Herring et al. [37], considering BMI and heart rate as primaries outcomes. This study compared the effects of MICT versus a control group in patients post bariatric surgery. The higher sample size was obtained for the outcome “heart rate.” A total of 17 participants per group were required to detect a significant decrease in the heart rate (effect size = 1.01). However, due to possible dropouts, 25 participants will be included in each group, totaling 75 volunteers.

### Recruitment {15}

Participant recruitment will occur weekly. A list of patients who are scheduled to have bariatric surgery will be provided by a staff member of the San Juan de Dios, Curicó’s Hospital, Chile. Participants who are interested and meet inclusion criteria will be invited to participate in the study after signing the informed consent.

## Assignment of interventions: allocation

### Sequence generation {16a}

#### Concealment mechanism {16b}

##### Implementation {16c}

**Fig. 2:**
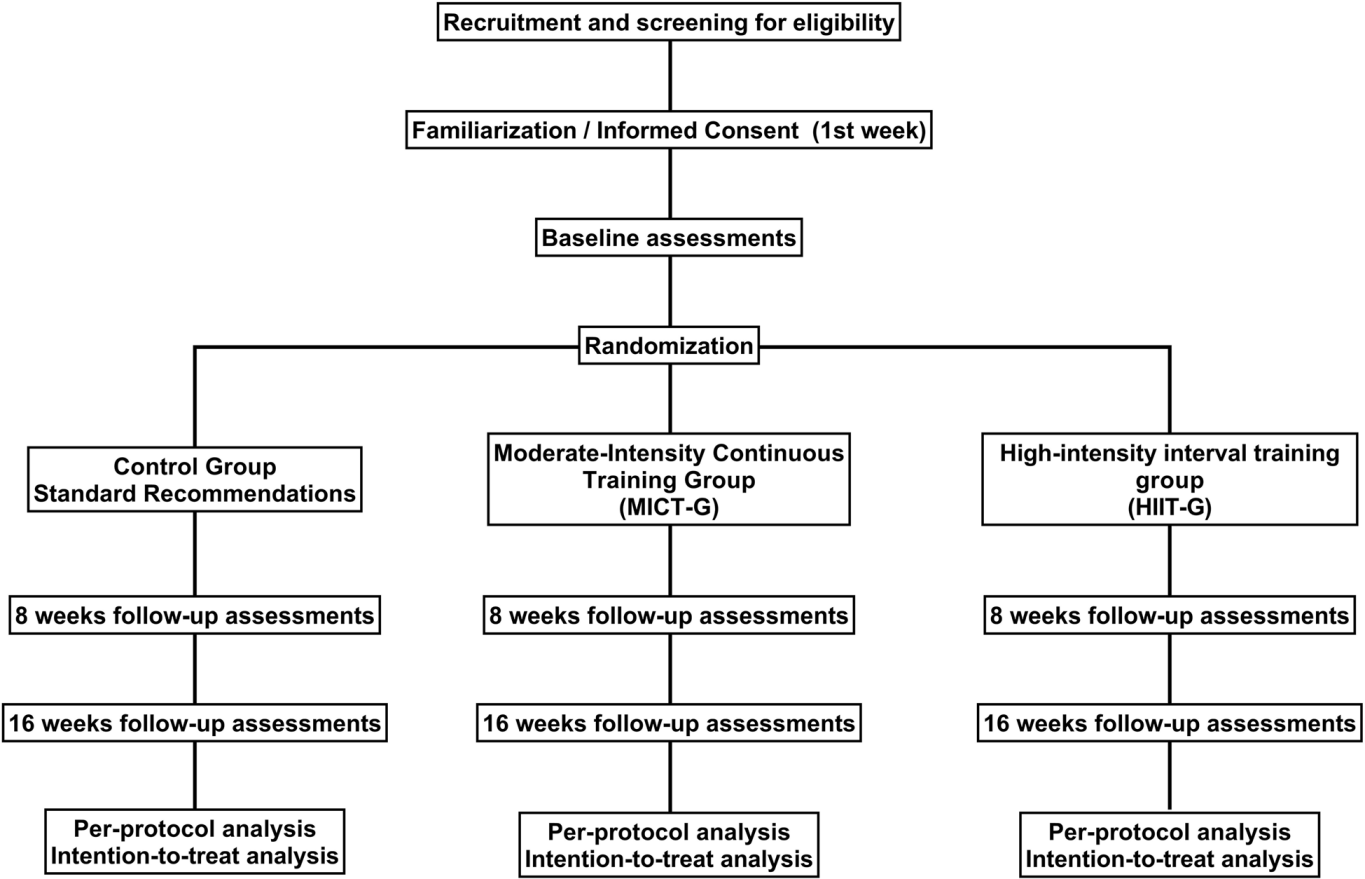
Study design flow chart

After baseline assessment, randomization will be performed using sealed opaque envelopes. Envelopes will contain a piece of paper containing the letters A, B, or C, corresponding to the three groups of the current study. An independent researcher (i.e., not involved in the current study) will organize the envelopes in numerical order and will keep them in a safe place until baseline assessments are concluded. Then, the envelope labeled with the same number as assigned to the participants when they were included in the study will be unsealed and informed to the leading research. Participants could be assigned to one of the three groups: (1) control group, (2) moderate-intensity continuous training group, and (3) high-intensity interval training group. Figure 2 shows the study design flow chart describing all the steps of the study (Fig. 2).

## Assignment of interventions: blinding

### Who will be blinded {17a}

This will be a single-blinded study where the outcome assessor (i.e., the researcher in charge of the evaluations) will have no information about the participants study group.

### Procedure for unblinding if needed {17b}

Not applicable, unblinding is not permissible in this trial.

## Data collection and management

### Plans for assessment and collection of outcomes {18a}

The assessments will be carried out at the institution’s Physical Medicine and Rehabilitation Service, and the intervention program will be carried out in the Service’s Adult Physical Therapy Gym. All the assessments will be conducted by a trained and experienced physical therapist (outcome assessor), which, prior the start of the study, was trained and familiarized with the evaluation protocol.

### Plans to promote participant retention and complete follow-up {18b}

A weekly phone call will be made to CG participants, asking about their health status and the indications for this group will be reinforced, in turn a text message will be sent to remind the evaluation date and time.

Participants who are in the training groups, in each session will be asked for feedback on their health status and will be sent a text message to remind them the training schedule.

In the event that any participant misses their training or evaluation, they will immediately proceed to call to ask about the reasons for not showing up.

### Data management {19}

All the information collected from this trial will always be protected and in the care of the lead research, who will assign a secure locker in his office where all documents of the investigation will be kept.

All electronic material will be duly stored and backed up in the researcher’s computer equipment with a safe password.

### Confidentiality {27}

In order to keep confidentiality after the assessments, the outcomes assessor will store the participants’ data separately from any identifying information and coded with a unique study ID. This ID will be linked to participant identity only within an encrypted, password-protected local database running on a secure host device maintained by outcome assessor and the study supervisor.

### Plans for collection, laboratory evaluation, and storage of biological specimens for genetic or molecular analysis in this trial/future use {33}

Not applicable, this trial does not have biological specimens.

## Statistical methods

### Statistical methods for primary and secondary outcomes {20a}

Data normality will be assessed with the Shapiro-Wilk test. Variance homogeneity and sphericity will be assessed with the Levene and Mauchly tests, respectively. For the primary and secondary outcomes that meet the assumptions for the analysis of variance (ANOVA), a mixed model ANOVA with Bonferroni correction will be used to assess the interaction between group (control group vs MICT vs HIIT) and time (baseline vs post 8-week intervention vs post 16-week intervention). Otherwise, data will be analyzed with the Wilcoxon test for within-group comparisons and the Mann-Whitney test for between-group comparisons using the Bonferroni’s correction. The significance level will be set at 5%. Cohen’s *d* will be calculated to determine the effect size. All analysis will be performed in SPSS 24.

### Interim analyses {21b}

Not applicable. Interim analyses will not be performed in the present study.

### Methods for additional analyses (e.g., subgroup analyses) {20b}

Not applicable. Additional analyses are not planned in the present study.

### Methods in analysis to handle protocol non-adherence and any statistical methods to handle missing data {20c}

Per-protocol and intention-to-treat analysis will be performed in order to account for the possible dropouts during the study. In the per-protocol analysis, we will include only the participants who attended at least 85% of the sessions and underwent the baseline and post intervention outcome assessments. For the intention-to-treat analysis, we will consider all the participants who underwent the baseline assessments and after being assigned to one of the study groups took part in at least one session. Missing data will be handled by multiple imputation method. Five imputed data sets will be obtained by multiple linear regression models for each variable. The final imputed value will be the arithmetic mean of the 5 data values created.

### Plans to give access to the full protocol, participant level-data and statistical code {31c}

Not applicable. Public access to the full protocol, data sets, and statistical code are not planned for this trial. However, this information might be available upon a reasonable request to the corresponding author keeping participants’ anonymity.

## Oversight and monitoring

### Composition of the coordinating center and trial steering committee {5d}

The center is coordinated by ATA and the leading researcher AHS. The trial will be directed by the principal investigators AHS and ARZ. No additional steering committee is considered for this study. All researchers will meet weekly to discuss the research progress and possible unforeseen events.

### Composition of the data monitoring committee, its role and reporting structure {21a}

No additional monitoring committee is considered for this study. The leading investigators (AHS and ARZ) will meet weekly with all the researchers involved in this study to discuss the research progress and any adverse event that arises during the procedures. Researchers are instructed to report any issue to the leading investigators immediately, who will then inform the board review committee from the San Juan de Dios, Curicó’s Hospital, and the Ethics Committee of the Universidad Católica del Maule, Talca, Chile, when appropriate.

### Adverse event reporting and harms {22}

In the end of each training session and evaluations, participants will be asked to report any complains and symptoms produced by the proposed activity. The outcome assessor and the lead researcher will be in charge to collect and record this information throughout the study. All complications and dropouts will be reported in the final manuscript.

### Frequency and plans for auditing trial conduct {23}

The trial conduct will be continuously monitored by the leading investigators. The San Juan de Dios, Curicó’s Hospital Review Board requires monthly report to get informed about the research’s progress and the occurrence of any adverse event. No extra auditing is considered for this study, unless requested by the Hospital’s Review Board or the Ethics Committee of the Universidad Católica del Maule.

### Plans for communicating important protocol amendments to relevant parties (e.g., trial participants, ethical committees) {25}

Protocol amendments need to be informed and approved by the San Juan de Dios, Curicó’s Hospital Review Board and the Ethics Committee of the Universidad Católica del Maule. Modifications will be updated at the clinicaltrials.gov by the leading investigator (AHS).

### Dissemination plans {31a}

Each participant will receive a full report with the results of their assessments. By the end of the study, the leading researcher will contact all the participants to provide the final results of the trial and to deliver educative material with information about healthy lifestyle and exercise recommendation. Secondary outcomes and preliminary results will be reported in local and international conferences. The final results will be submitted to a peer-reviewed indexed scientific journal within the 5 years after the last participant was enrolled.

## Discussion

The objective of this study will be to investigate the effects of 16 weeks of MICT and HIIT on body composition, cardiopulmonary function, and perceived quality of life in bariatric surgery patients.

MICT has been proven effective for increasing fat mass loss and decreasing lean mass loss after bariatric surgery, as well as for improving control of glucose homeostasis and cardiovascular capacity [21, 22]. On the other hand, HITT protocols, which involve repeating intense efforts that last a few seconds separated by short periods of recovery, have shown to promote beneficial changes in patients with lifestyle-induced chronic diseases [38]. Also, HIIT improves cardiorespiratory fitness and insulin sensitivity in obese patients and is well tolerated by overweight and insulin-resistant people [25–27]. However, to the best of our knowledge, no studies have compared MICT and HITT protocols in bariatric surgery patients.

It should be also pointed out that besides relevant outcomes, such as body composition and functional capacity, this study consider the assessment of the cardiac autonomic control, which will provide important information about the effectiveness of both methods in a cardiovascular marker related to the risk of cardiovascular events and mortality [39, 40]. Determining which exercise strategy better suits this population and is more effective regarding these outcomes is relevant for bariatric surgery management and formulating recommendations.

Another strength of our study is the presence of a control group that follows current Chilean recommendations after bariatric surgery. If our hypothesis is confirmed, this exploratory pilot trial could be a start point to new recommendations about supervised exercise after bariatric surgery.

## Trial status

Recruiting.

Version 2. January 21, 2020.

Date recruitment began: December 2, 2019.

Approximate date when recruitment will be completed: December 31, 2022.

## Data Availability

After study publication, the data and materials will be available upon a reasonable request to the corresponding author.
